# 17-Acet­oxy­mulinic acid

**DOI:** 10.1107/S1600536810032952

**Published:** 2010-08-28

**Authors:** Iván Brito, Jorge Bórquez, Joselyn Albanez, Michael Bolte, Luis Manuel Peña-Rodríguez

**Affiliations:** a Departamento de Química, Facultad de Ciencias Básicas, Universidad de Antofagasta, Casilla 170, Antofagasta, Chile; b Institut für Anorganische Chemie der Goethe-Universität Frankfurt, Max-von-Laue-Strasse 7, D-60438 Frankfurt am Main, Germany; c Unidad de Biotecnología, Centro de Investigacion Científica de Yucatán, Calle 43, N° 130, Colonia Chuburná, Mérida, Yucatán 97200, Mexico

## Abstract

The title compound, [systematic name: 5a-acet­oxy­methyl-3-isopropyl-8-methyl-1,2,3,3a,4,5,5a,6,7,10,10a,10b-dodeca­hydro-7,10-*endo*-epidi­oxy­cyclo­hepta­[*e*]indene-3a-carb­oxy­lic acid], C_22_H_32_O_6_ (I), is closely related to methyl 5a-acet­oxy­methyl-3-isopropyl-8-methyl-1,2,3,3a,4,5,5a,6,7,10,10a,10b-dodeca­hydro-7,10-*endo*-epidi­oxy­cyclo­hepta­[*e*]indene-3a-carboxyl­ate, (II) [Brito *et al.*, (2008 ▶). *Acta Cryst.* E**64**, o1209]. There are two mol­ecules in the asymmetric unit, which are linked by two strong intra­molecular O—H⋯O hydrogen bonds with graph-set motif *R*_2_^2^(8). In both (I) and (II), the conformation of the three fused rings are almost identical. The five-membered ring has an envelope conformation, the six-membered ring has a chair conformation and the seven-membered ring has a boat conformation. The most obvious differences between the two compounds is the observed disorder of the acet­oxy­methyl fragments in both mol­ecules of the asymmetric unit of (I). This disorder is not observed in (II). The crystal structure and the molecular conformation is stabilized by intermolecular C—H⋯O hydrogen bonds. The ability to form hydrogen bonds is different in the two compounds. The crystal studied was a non-merohedral twin, the ratio of the twin components being 0.28 (1):0.72 (1)

## Related literature

For related literature on Mulinane diterpenes, see: Munizaga & Gunkel (1958 ▶); Araya *et al.* (2003 ▶); Loyola *et al.* (1990 ▶, 2004 ▶). For a related structure, see: Brito *et al.* (2008 ▶). For ring conformations, see: Cremer & Pople (1975 ▶). For hydrogen-bond patterns, see: Bernstein *et al.* (1995 ▶). For bond-length data, see: Allen *et al.* (1987 ▶).
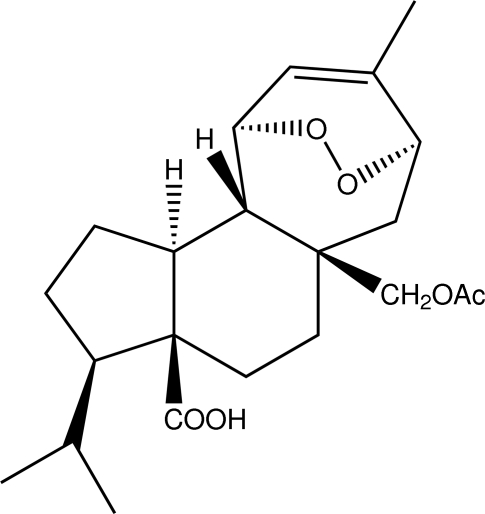


## Experimental

### 

#### Crystal data

C_22_H_32_O_6_
*M* *_r_* = 392.48
Monoclinic, 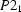

*a* = 11.9171 (12) Å
*b* = 7.3523 (4) Å
*c* = 23.679 (2) Å
β = 92.775 (8)°
*V* = 2072.3 (3) Å^3^
*Z* = 4
Mo *K*α radiation
μ = 0.09 mm^−1^
*T* = 173 K
0.24 × 0.22 × 0.22 mm

#### Data collection

Stoe IPDS II two-circle diffractometer
12732 measured reflections
3948 independent reflections
2516 reflections with *I* > 2σ(*I*)
*R*_int_ = 0.085

#### Refinement

*R*[*F* ^2^ > 2σ(*F* ^2^)] = 0.055
*wR*(*F* ^2^) = 0.137
*S* = 0.90
3948 reflections
507 parameters
1 restraint
H-atom parameters constrained
Δρ_max_ = 0.27 e Å^−3^
Δρ_min_ = −0.25 e Å^−3^

### 

Data collection: *X-AREA* (Stoe & Cie, 2001 ▶); cell refinement: *X-AREA*; data reduction: *X-AREA*; program(s) used to solve structure: *SHELXS97* (Sheldrick, 2008 ▶); program(s) used to refine structure: *SHELXL97* (Sheldrick, 2008 ▶); molecular graphics: *XP* (Sheldrick, 2008 ▶); software used to prepare material for publication: *SHELXL97*.

## Supplementary Material

Crystal structure: contains datablocks I, global. DOI: 10.1107/S1600536810032952/om2352sup1.cif

Structure factors: contains datablocks I. DOI: 10.1107/S1600536810032952/om2352Isup2.hkl

Additional supplementary materials:  crystallographic information; 3D view; checkCIF report

## Figures and Tables

**Table 1:** Hydrogen-bond geometry (Å, °)

*D*—H⋯*A*	*D*—H	H⋯*A*	*D*⋯*A*	*D*—H⋯*A*
O6—H6⋯O5*A*	0.84	1.90	2.714 (5)	162
O6*A*—H6*A*⋯O5	0.84	1.88	2.690 (5)	161
C5*B*—H5*B*1⋯O4*A*^i^	0.99	2.37	3.257 (14)	149
C10—H10⋯O2^ii^	1.00	2.34	3.259 (9)	152
C10*A*—H10*A*⋯O5	1.00	2.50	3.134 (9)	121
C10*C*—H10*C*⋯O2*A*^iii^	1.00	2.46	3.350 (8)	147
C10*D*—H10*D*⋯O5*A*	1.00	2.51	3.151 (8)	122

Symmetry codes: (i) 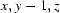; (ii) 
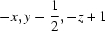
; (iii) 
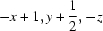
.

**Fig. 1.**
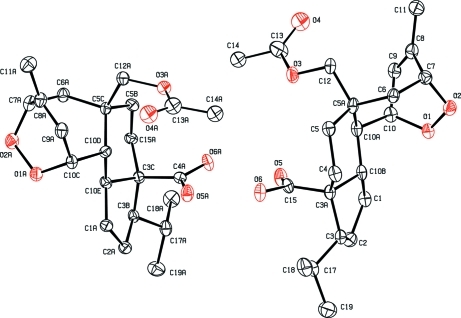
Perspective view of the two molecules of (I) in the asymmetric unit. Displacement ellipsoids are drawn at the 30% probability level. H atoms have been omitted for clarity.

**Fig. 2.**
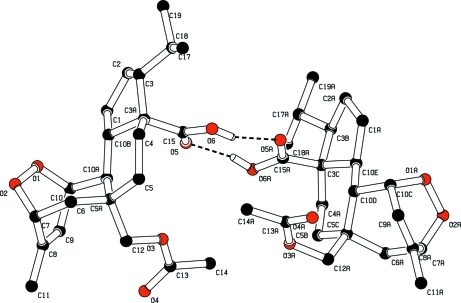
Perspective view of the two molecules in the asymmetric unit, showing the intramolecular O—H···O hydrogen bond. H atoms bonded to C atoms have been omitted for clarity.

## supplementary crystallographic information

### Comment

The title compound (I) is know as 17-acetoxymulinic acid and was isolated from Mulinum crassifolium (Apiaceae). Mulinum crassifolium is a 15 cm small shrub, which grows in the north of Chile at altitudes above 4000 m. This plant, commonly known as chuquican, susurco or espinilla is used in the folk medicine, principally against diabetes, and bronchial (caugh) and intestinal disorders (Munizaga *et al.*, 1958). Mulinane diterpenes exhibit antiplasmodial (Loyola *et al.*, 2004) and anti-Tripanosomacruzi (Araya *et al.*, 2003) activities. We have undertaken the X-ray crystal-structure determination of (I) in order to establish its molecular conformation and relative stereochemistry. We are not able to determine the absolute stereochemistry by X-ray methods and the configuration shown here was chosen to be in accord with that reported in previous chemical studies (Loyola *et al.*, 1990). The structure consists of a mulinane skeleton, and the isopropyl, acetyloxymethyl and carboxylic groups at C3, C5A and C3A are β-oriented, respectively, whereas the *endo*-peroxide group is α-oriented. The molecular dimensions of the title compound are within normal ranges (Allen *et al.*, 1987). The cyclopentane (A), cyclohexane (B) and cycloheptene (C) rings are in an envelope, chair and boat conformation, respectively [Q_2_ = 0.4233 (8) Å, φ_2 _= 176.0 (10)° for ring A (mean); Q_T_ = 0.553 (8) Å, θ = 161.3 (8)°, φ = 238 (2)° for ring B (mean); Q_T_ = 1.230 (4) Å, φ_2 _= 169.9 (2)°, for ring C(mean)] (Cremer & Pople, 1975), Fig.1. The A and B and B and C rings are *trans* and *cis*-fused respectively. The crystal structure and the molecular conformation is stabilized by three and eleven C—H···O hydrogen bonds respectively. The two molecules in the asymmetric unit are linked by two strong O—H···O intramolecular hydrogen bond with set graph-motif *R*^2^_2_(8), Fig. 2. (Bernstein *et al.*, 1995).

### Experimental

Dried and finely powdered aerial parts of Mulinum crassifolium (630 g) were extracted with petroleum ether at room temperature. The concentrated petroleum extract was fractionated on silica gel column with hexane-ethyl acetate mixtures of increasing polarity as elution solvents. The fraction (355 mg) eluted was further separated and purified by silica gel chromatography to give 52.2 mg of 17-acetoxymulinic acid. Recrystallization from hexane-ethyl acetate (3:1) at room temperature afforded colourless crystals suitable for X-ray diffraction analysis.

### Refinement

There is disorder in the acetyloxymethyl fragment of both molecules of the asymmetric unit. O4 and C14 are disordered over two orientations with occupancy factors of 0.53 (2)/0.47 (2) for O4/O4' and C14/C14'. O3A, O4A, C13A, C14A are disordered over two orientations with occupancy factors of 0.612 (9)/0.388 (9) for O3A/O3", O4A/O4", C13A/C13" and C14A/C14". respectively. These six disordered atoms are refined isotropically, while all other non-hydrogen atoms are refined anisotropically. All H atoms were placed in idealized positions with d(C—H,O) = 0.99 (CH_2_), 0.98 (CH_3_), 1.00 (CH) and 0.84 Å (OH), refined using a riding model with *U*_iso_(H) fixed at 1.5 *U*_eq_(C,O) for CH_3 _and OH and 1.2 *U*_eq_(C) for CH_2_ and CH. The crystal studied was a non-merohedral twin, the ratio of the twin components being 0.28 (1): 0.72 (1)

### Crystal data

**Table:**

C_22_H_32_O_6_	*F*(000) = 848
*M**_r_* = 392.48	*D*_x_ = 1.258 Mg m^−^^3^
Monoclinic, *P*2_1_	Mo *K*α radiation, λ = 0.71073 Å
Hall symbol: P 2yb	Cell parameters from 7317 reflections
*a* = 11.9171 (12) Å	θ = 2.4–24.8°
*b* = 7.3523 (4) Å	µ = 0.09 mm^−^^1^
*c* = 23.679 (2) Å	*T* = 173 K
β = 92.775 (8)°	Block, colourless
*V* = 2072.3 (3) Å^3^	0.24 × 0.22 × 0.22 mm
*Z* = 4	

### Data collection

**Table:**

Stoe IPDS II two-circle diffractometer	2516 reflections with *I* > 2σ(*I*)
Radiation source: fine-focus sealed tube	*R*_int_ = 0.085
graphite	θ_max_ = 25.1°, θ_min_ = 2.4°
ω scans	*h* = −14→14
12732 measured reflections	*k* = −8→8
3948 independent reflections	*l* = −28→28

### Refinement

**Table:**

Refinement on *F*^2^	Secondary atom site location: difference Fourier map
Least-squares matrix: full	Hydrogen site location: inferred from neighbouring sites
*R*[*F*^2^ > 2σ(*F*^2^)] = 0.055	H-atom parameters constrained
*wR*(*F*^2^) = 0.137	*w* = 1/[σ^2^(*F*_o_^2^) + (0.0735*P*)^2^] where *P* = (*F*_o_^2^ + 2*F*_c_^2^)/3
*S* = 0.90	(Δ/σ)_max_ = 0.002
3948 reflections	Δρ_max_ = 0.27 e Å^−^^3^
507 parameters	Δρ_min_ = −0.25 e Å^−^^3^
1 restraint	Extinction correction: *SHELXL97* (Sheldrick, 2008), Fc^*^=kFc[1+0.001xFc^2^λ^3^/sin(2θ)]^-1/4^
Primary atom site location: structure-invariant direct methods	Extinction coefficient: 0.024 (3)

### Special details

**Table:**

Geometry. All s.u.'s (except the s.u. in the dihedral angle between two l.s. planes) are estimated using the full covariance matrix. The cell s.u.'s are taken into account individually in the estimation of s.u.'s in distances, angles and torsion angles; correlations between s.u.'s in cell parameters are only used when they are defined by crystal symmetry. An approximate (isotropic) treatment of cell s.u.'s is used for estimating s.u.'s involving l.s. planes.
Refinement. Refinement of F^2^ against ALL reflections. The weighted R-factor wR and goodness of fit S are based on F^2^, conventional R-factors R are based on F, with F set to zero for negative F^2^. The threshold expression of F^2^ > 2σ(F^2^) is used only for calculating R-factors(gt) etc. and is not relevant to the choice of reflections for refinement. R-factors based on F^2^ are statistically about twice as large as those based on F, and R- factors based on ALL data will be even larger.

### Fractional atomic coordinates and isotropic or equivalent isotropic displacement parameters (Å^2^)

**Table:**

	*x*	*y*	*z*	*U*_iso_*/*U*_eq_	Occ. (<1)
O1	0.0330 (3)	0.5360 (6)	0.50763 (16)	0.0412 (10)	
O2	0.0844 (3)	0.6426 (5)	0.55530 (16)	0.0410 (10)	
O3	0.3854 (3)	0.5138 (6)	0.39357 (16)	0.0481 (11)	
O4	0.4564 (11)	0.2699 (17)	0.4403 (4)	0.064 (4)*	0.53 (2)
O4'	0.5134 (15)	0.331 (2)	0.4317 (6)	0.081 (6)*	0.47 (2)
O5	0.1365 (3)	0.5201 (5)	0.29474 (17)	0.0449 (11)	
O6	0.1908 (3)	0.7900 (6)	0.26552 (17)	0.0437 (11)	
H6	0.2232	0.7251	0.2421	0.052*	
C1	−0.0445 (5)	0.5462 (8)	0.3769 (2)	0.0380 (14)	
H1A	−0.0967	0.5256	0.4075	0.046*	
H1B	−0.0195	0.4268	0.3629	0.046*	
C2	−0.1020 (5)	0.6568 (9)	0.3288 (3)	0.0426 (15)	
H2A	−0.1819	0.6780	0.3365	0.051*	
H2B	−0.0987	0.5900	0.2926	0.051*	
C3	−0.0393 (4)	0.8399 (8)	0.3252 (2)	0.0343 (13)	
H3	−0.0711	0.9222	0.3540	0.041*	
C3A	0.0828 (4)	0.7869 (8)	0.3480 (2)	0.0320 (13)	
C4	0.1570 (5)	0.9381 (8)	0.3734 (2)	0.0380 (14)	
H4A	0.1160	1.0052	0.4022	0.046*	
H4B	0.1765	1.0250	0.3435	0.046*	
C5	0.2653 (4)	0.8535 (8)	0.4009 (2)	0.0365 (13)	
H5A	0.3091	0.9525	0.4198	0.044*	
H5B	0.3107	0.8065	0.3701	0.044*	
C5A	0.2531 (5)	0.6980 (8)	0.4445 (2)	0.0354 (14)	
C6	0.2356 (5)	0.7812 (8)	0.5026 (2)	0.0335 (13)	
H6C	0.1756	0.8739	0.4978	0.040*	
H6D	0.3055	0.8464	0.5146	0.040*	
C7	0.2048 (5)	0.6554 (9)	0.5519 (2)	0.0385 (14)	
H7	0.2340	0.7153	0.5876	0.046*	
C8	0.2514 (5)	0.4650 (9)	0.5526 (3)	0.0417 (15)	
C9	0.2025 (5)	0.3582 (8)	0.5131 (2)	0.0384 (14)	
H9	0.2237	0.2344	0.5095	0.058*	
C10A	0.1563 (4)	0.5629 (8)	0.4263 (2)	0.0346 (14)	
H10A	0.1867	0.4793	0.3975	0.042*	
C10B	0.0552 (4)	0.6587 (8)	0.3982 (2)	0.0324 (13)	
H10B	0.0259	0.7403	0.4278	0.039*	
C10	0.1127 (5)	0.4408 (8)	0.4747 (2)	0.0376 (14)	
H10	0.0712	0.3374	0.4559	0.045*	
C11	0.3453 (5)	0.4133 (10)	0.5925 (3)	0.0508 (17)	
H11A	0.3617	0.2836	0.5883	0.076*	
H11B	0.3242	0.4374	0.6313	0.076*	
H11C	0.4120	0.4848	0.5845	0.076*	
C12	0.3707 (4)	0.6057 (9)	0.4477 (2)	0.0379 (14)	
H12A	0.4302	0.6982	0.4545	0.045*	
H12B	0.3756	0.5165	0.4790	0.045*	
C13	0.4386 (7)	0.3625 (11)	0.3940 (3)	0.065 (2)	
C14	0.4594 (15)	0.290 (2)	0.3359 (6)	0.049 (4)*	0.53 (2)
H14A	0.4997	0.1739	0.3394	0.073*	0.53 (2)
H14B	0.5047	0.3774	0.3157	0.073*	0.53 (2)
H14C	0.3874	0.2711	0.3150	0.073*	0.53 (2)
C14'	0.4201 (16)	0.260 (2)	0.3396 (6)	0.043 (4)*	0.47 (2)
H14D	0.4620	0.1450	0.3419	0.064*	0.47 (2)
H14E	0.4465	0.3329	0.3083	0.064*	0.47 (2)
H14F	0.3399	0.2340	0.3331	0.064*	0.47 (2)
C15	0.1416 (5)	0.6869 (8)	0.3009 (2)	0.0345 (13)	
C17	−0.0562 (5)	0.9350 (9)	0.2681 (2)	0.0400 (14)	
H17	−0.0242	0.8549	0.2388	0.048*	
C18	0.0010 (6)	1.1199 (9)	0.2651 (3)	0.0520 (17)	
H18A	−0.0127	1.1717	0.2273	0.078*	
H18B	0.0821	1.1054	0.2729	0.078*	
H18C	−0.0294	1.2016	0.2933	0.078*	
C19	−0.1820 (5)	0.9598 (12)	0.2528 (3)	0.065 (2)	
H19A	−0.1914	1.0206	0.2160	0.098*	
H19B	−0.2162	1.0340	0.2817	0.098*	
H19C	−0.2187	0.8405	0.2507	0.098*	
O1A	0.5412 (3)	0.5914 (5)	0.01076 (16)	0.0380 (10)	
O2A	0.6361 (3)	0.4646 (5)	0.00595 (17)	0.0423 (10)	
O3A	0.5649 (5)	0.5736 (11)	0.2327 (3)	0.0378 (19)*	0.612 (9)
O3"	0.5776 (9)	0.6638 (19)	0.2175 (5)	0.041 (3)*	0.388 (9)
O4A	0.5833 (6)	0.8545 (12)	0.2002 (3)	0.064 (3)*	0.612 (9)
O4"	0.6087 (9)	0.5455 (18)	0.3025 (5)	0.058 (4)*	0.388 (9)
O5A	0.2604 (3)	0.6085 (5)	0.17374 (17)	0.0436 (11)	
O6A	0.2480 (3)	0.3393 (6)	0.21618 (16)	0.0408 (10)	
H6A	0.2009	0.3945	0.2351	0.049*	
C1A	0.2944 (4)	0.5789 (7)	0.0507 (2)	0.0322 (13)	
H1A1	0.3159	0.5998	0.0113	0.039*	
H1A2	0.2868	0.6981	0.0696	0.039*	
C2A	0.1831 (4)	0.4711 (8)	0.0514 (2)	0.0348 (13)	
H2A1	0.1535	0.4465	0.0124	0.042*	
H2A2	0.1261	0.5405	0.0715	0.042*	
C3B	0.2116 (4)	0.2898 (7)	0.0829 (2)	0.0292 (12)	
H3B	0.2387	0.2036	0.0539	0.035*	
C3C	0.3159 (4)	0.3388 (7)	0.1226 (2)	0.0283 (12)	
C4A	0.3948 (4)	0.1871 (8)	0.1412 (2)	0.0328 (13)	
H4A1	0.4175	0.1177	0.1078	0.039*	
H4A2	0.3562	0.1028	0.1664	0.039*	
C5B	0.4995 (4)	0.2683 (8)	0.1726 (2)	0.0357 (14)	
H5B1	0.5522	0.1672	0.1818	0.043*	
H5B2	0.4757	0.3185	0.2090	0.043*	
C5C	0.5658 (4)	0.4192 (8)	0.1425 (2)	0.0306 (13)	
C6A	0.6466 (4)	0.3295 (8)	0.1010 (2)	0.0350 (14)	
H6A1	0.7064	0.2667	0.1240	0.042*	
H6A2	0.6036	0.2345	0.0796	0.042*	
C7A	0.7043 (5)	0.4492 (8)	0.0577 (2)	0.0384 (14)	
H7A	0.7747	0.3846	0.0481	0.046*	
C8A	0.7384 (4)	0.6337 (8)	0.0780 (2)	0.0378 (14)	
C9A	0.6526 (5)	0.7514 (9)	0.0810 (2)	0.0395 (15)	
H9A	0.6642	0.8739	0.0926	0.059*	
C10C	0.5386 (4)	0.6802 (8)	0.0652 (2)	0.0329 (13)	
H10C	0.4884	0.7885	0.0599	0.039*	
C10D	0.4843 (4)	0.5564 (8)	0.1100 (2)	0.0296 (12)	
H10D	0.4554	0.6401	0.1393	0.035*	
C10E	0.3815 (4)	0.4619 (8)	0.0825 (2)	0.0302 (12)	
H10E	0.4115	0.3781	0.0536	0.036*	
C11A	0.8593 (5)	0.6750 (10)	0.0944 (3)	0.0508 (16)	
H11D	0.8657	0.8008	0.1079	0.076*	
H11E	0.9051	0.6592	0.0615	0.076*	
H11F	0.8858	0.5920	0.1246	0.076*	
C12A	0.6384 (4)	0.5102 (9)	0.1902 (2)	0.0384 (14)	
H12C	0.6930	0.4216	0.2070	0.046*	
H12D	0.6806	0.6136	0.1749	0.046*	
C13A	0.5438 (10)	0.7518 (19)	0.2333 (5)	0.056 (3)*	0.612 (9)
C13"	0.5646 (12)	0.659 (2)	0.2753 (7)	0.040 (4)*	0.388 (9)
C14A	0.4736 (11)	0.819 (2)	0.2780 (6)	0.051 (4)*	0.612 (9)
H14G	0.4453	0.7151	0.2992	0.076*	0.612 (9)
H14H	0.4102	0.8876	0.2611	0.076*	0.612 (9)
H14I	0.5185	0.8975	0.3037	0.076*	0.612 (9)
C14"	0.4819 (19)	0.771 (3)	0.2932 (10)	0.059 (7)*	0.388 (9)
H14J	0.4592	0.8555	0.2628	0.089*	0.388 (9)
H14K	0.5103	0.8394	0.3264	0.089*	0.388 (9)
H14L	0.4170	0.6977	0.3032	0.089*	0.388 (9)
C15A	0.2741 (4)	0.4393 (8)	0.1734 (2)	0.0292 (12)	
C17A	0.1088 (5)	0.1993 (8)	0.1083 (2)	0.0371 (14)	
H17A	0.0822	0.2807	0.1387	0.044*	
C18A	0.1343 (5)	0.0147 (8)	0.1342 (3)	0.0476 (16)	
H18D	0.0662	−0.0345	0.1502	0.071*	
H18E	0.1933	0.0271	0.1643	0.071*	
H18F	0.1598	−0.0681	0.1051	0.071*	
C19A	0.0123 (5)	0.1781 (10)	0.0630 (3)	0.0506 (17)	
H19D	−0.0047	0.2966	0.0457	0.076*	
H19E	−0.0546	0.1315	0.0807	0.076*	
H19F	0.0349	0.0928	0.0339	0.076*	

### Atomic displacement parameters (Å^2^)

**Table:**

	*U*^11^	*U*^22^	*U*^33^	*U*^12^	*U*^13^	*U*^23^
O1	0.043 (2)	0.048 (2)	0.033 (2)	0.000 (2)	0.0080 (18)	0.003 (2)
O2	0.046 (2)	0.039 (2)	0.040 (2)	0.0040 (19)	0.0085 (18)	−0.002 (2)
O3	0.062 (3)	0.050 (3)	0.034 (2)	0.017 (2)	0.0125 (19)	−0.001 (2)
O5	0.066 (3)	0.030 (3)	0.040 (2)	−0.008 (2)	0.016 (2)	−0.008 (2)
O6	0.054 (2)	0.039 (2)	0.040 (2)	−0.005 (2)	0.022 (2)	−0.005 (2)
C1	0.045 (3)	0.035 (3)	0.035 (3)	−0.008 (3)	0.008 (3)	−0.004 (3)
C2	0.039 (3)	0.046 (4)	0.043 (3)	−0.002 (3)	0.003 (3)	0.001 (3)
C3	0.041 (3)	0.033 (3)	0.030 (3)	0.000 (3)	0.008 (2)	−0.002 (3)
C3A	0.038 (3)	0.028 (3)	0.030 (3)	−0.004 (2)	0.008 (2)	−0.002 (3)
C4	0.050 (3)	0.028 (3)	0.037 (3)	−0.010 (3)	0.009 (3)	0.000 (3)
C5	0.039 (3)	0.035 (3)	0.036 (3)	−0.007 (3)	0.005 (3)	−0.002 (3)
C5A	0.045 (3)	0.027 (3)	0.035 (3)	0.003 (3)	0.012 (3)	−0.009 (3)
C6	0.038 (3)	0.028 (3)	0.035 (3)	0.001 (2)	0.009 (2)	−0.001 (3)
C7	0.042 (3)	0.038 (3)	0.035 (3)	0.002 (3)	0.002 (3)	−0.002 (3)
C8	0.044 (3)	0.040 (4)	0.042 (3)	0.000 (3)	0.012 (3)	0.002 (3)
C9	0.050 (3)	0.027 (3)	0.040 (3)	0.000 (3)	0.013 (3)	0.007 (3)
C10A	0.045 (3)	0.027 (3)	0.033 (3)	−0.010 (3)	0.014 (3)	−0.004 (3)
C10B	0.041 (3)	0.031 (3)	0.026 (3)	−0.006 (3)	0.010 (2)	−0.007 (3)
C10	0.048 (3)	0.027 (3)	0.039 (3)	−0.003 (3)	0.006 (3)	−0.004 (3)
C11	0.055 (4)	0.050 (4)	0.048 (4)	0.008 (3)	0.005 (3)	0.010 (3)
C12	0.040 (3)	0.042 (4)	0.033 (3)	−0.003 (3)	0.013 (2)	−0.003 (3)
C13	0.097 (6)	0.049 (5)	0.047 (4)	0.027 (4)	−0.012 (4)	−0.013 (4)
C15	0.041 (3)	0.034 (4)	0.028 (3)	−0.007 (3)	0.008 (2)	0.006 (3)
C17	0.050 (3)	0.039 (3)	0.031 (3)	−0.006 (3)	0.006 (3)	−0.003 (3)
C18	0.073 (4)	0.037 (4)	0.047 (4)	−0.006 (3)	0.012 (3)	0.010 (3)
C19	0.055 (4)	0.079 (6)	0.061 (4)	−0.004 (4)	−0.002 (3)	0.016 (4)
O1A	0.039 (2)	0.039 (2)	0.037 (2)	0.0012 (18)	0.0062 (17)	0.001 (2)
O2A	0.052 (2)	0.034 (2)	0.041 (2)	0.0058 (19)	0.0062 (19)	−0.009 (2)
O5A	0.058 (3)	0.032 (2)	0.042 (2)	0.005 (2)	0.017 (2)	−0.002 (2)
O6A	0.059 (2)	0.039 (2)	0.026 (2)	−0.002 (2)	0.0114 (18)	0.0011 (19)
C1A	0.035 (3)	0.030 (3)	0.032 (3)	0.003 (2)	0.002 (2)	0.005 (3)
C2A	0.035 (3)	0.037 (3)	0.033 (3)	0.003 (2)	0.003 (2)	0.008 (3)
C3B	0.037 (3)	0.026 (3)	0.025 (3)	0.003 (2)	0.007 (2)	0.002 (2)
C3C	0.036 (3)	0.024 (3)	0.025 (3)	0.006 (2)	0.004 (2)	0.001 (2)
C4A	0.032 (3)	0.036 (3)	0.030 (3)	0.002 (2)	−0.001 (2)	0.008 (3)
C5B	0.040 (3)	0.035 (3)	0.032 (3)	0.003 (3)	0.002 (2)	0.011 (3)
C5C	0.032 (3)	0.033 (3)	0.027 (3)	0.004 (2)	0.005 (2)	−0.003 (3)
C6A	0.039 (3)	0.031 (3)	0.035 (3)	0.003 (3)	0.006 (3)	−0.004 (3)
C7A	0.040 (3)	0.035 (3)	0.041 (3)	0.000 (3)	0.012 (3)	−0.003 (3)
C8A	0.036 (3)	0.039 (4)	0.039 (3)	−0.004 (3)	0.008 (3)	0.000 (3)
C9A	0.042 (3)	0.037 (3)	0.040 (3)	−0.005 (3)	0.009 (3)	−0.004 (3)
C10C	0.039 (3)	0.031 (3)	0.030 (3)	−0.001 (2)	0.009 (2)	0.000 (3)
C10D	0.028 (3)	0.029 (3)	0.032 (3)	0.002 (2)	0.003 (2)	0.002 (3)
C10E	0.036 (3)	0.028 (3)	0.026 (3)	0.001 (2)	0.001 (2)	0.003 (3)
C11A	0.043 (3)	0.049 (4)	0.060 (4)	−0.005 (3)	0.001 (3)	−0.009 (4)
C12A	0.040 (3)	0.042 (4)	0.033 (3)	0.007 (3)	0.001 (3)	−0.006 (3)
C15A	0.030 (3)	0.029 (3)	0.029 (3)	−0.001 (2)	−0.001 (2)	0.000 (3)
C17A	0.045 (3)	0.032 (3)	0.035 (3)	−0.005 (3)	0.012 (3)	0.003 (3)
C18A	0.055 (4)	0.030 (3)	0.059 (4)	−0.007 (3)	0.009 (3)	0.010 (3)
C19A	0.040 (3)	0.058 (4)	0.053 (4)	−0.016 (3)	−0.006 (3)	0.010 (4)

### Geometric parameters (Å, °)

**Table:**

O1—C10	1.440 (6)	O1A—O2A	1.474 (5)
O1—O2	1.482 (6)	O2A—C7A	1.443 (7)
O2—C7	1.444 (6)	O3A—C13A	1.334 (16)
O3—C13	1.280 (8)	O3A—C12A	1.443 (8)
O3—C12	1.466 (7)	O3"—C13"	1.385 (19)
O4—C13	1.300 (13)	O3"—C12A	1.504 (14)
O4'—C13	1.252 (15)	O4A—C13A	1.200 (13)
O5—C15	1.236 (7)	O4"—C13"	1.162 (19)
O6—C15	1.291 (6)	O5A—C15A	1.255 (7)
O6—H6	0.8400	O6A—C15A	1.301 (6)
C1—C10B	1.514 (8)	O6A—H6A	0.8400
C1—C2	1.535 (8)	C1A—C10E	1.520 (7)
C1—H1A	0.9900	C1A—C2A	1.546 (7)
C1—H1B	0.9900	C1A—H1A1	0.9900
C2—C3	1.544 (8)	C1A—H1A2	0.9900
C2—H2A	0.9900	C2A—C3B	1.556 (8)
C2—H2B	0.9900	C2A—H2A1	0.9900
C3—C17	1.525 (8)	C2A—H2A2	0.9900
C3—C3A	1.577 (8)	C3B—C17A	1.542 (7)
C3—H3	1.0000	C3B—C3C	1.564 (8)
C3A—C4	1.526 (8)	C3B—H3B	1.0000
C3A—C15	1.533 (7)	C3C—C4A	1.510 (7)
C3A—C10B	1.564 (8)	C3C—C15A	1.517 (7)
C4—C5	1.548 (9)	C3C—C10E	1.550 (7)
C4—H4A	0.9900	C4A—C5B	1.543 (8)
C4—H4B	0.9900	C4A—H4A1	0.9900
C5—C5A	1.553 (8)	C4A—H4A2	0.9900
C5—H5A	0.9900	C5B—C5C	1.555 (7)
C5—H5B	0.9900	C5B—H5B1	0.9900
C5A—C6	1.529 (7)	C5B—H5B2	0.9900
C5A—C12	1.555 (8)	C5C—C12A	1.542 (8)
C5A—C10A	1.567 (8)	C5C—C6A	1.556 (7)
C6—C7	1.548 (8)	C5C—C10D	1.575 (8)
C6—H6C	0.9900	C6A—C7A	1.538 (8)
C6—H6D	0.9900	C6A—H6A1	0.9900
C7—C8	1.505 (9)	C6A—H6A2	0.9900
C7—H7	1.0000	C7A—C8A	1.489 (8)
C8—C9	1.332 (9)	C7A—H7A	1.0000
C8—C11	1.480 (9)	C8A—C9A	1.344 (8)
C9—C10	1.499 (8)	C8A—C11A	1.506 (8)
C9—H9	0.9500	C9A—C10C	1.486 (8)
C10A—C10B	1.521 (8)	C9A—H9A	0.9500
C10A—C10	1.563 (7)	C10C—C10D	1.562 (7)
C10A—H10A	1.0000	C10C—H10C	1.0000
C10B—H10B	1.0000	C10D—C10E	1.526 (8)
C10—H10	1.0000	C10D—H10D	1.0000
C11—H11A	0.9800	C10E—H10E	1.0000
C11—H11B	0.9800	C11A—H11D	0.9800
C11—H11C	0.9800	C11A—H11E	0.9800
C12—H12A	0.9900	C11A—H11F	0.9800
C12—H12B	0.9900	C12A—H12C	0.9900
C13—C14	1.507 (15)	C12A—H12D	0.9900
C13—C14'	1.501 (16)	C13A—C14A	1.466 (18)
C14—H14A	0.9800	C13"—C14"	1.37 (3)
C14—H14B	0.9800	C14A—H14G	0.9800
C14—H14C	0.9800	C14A—H14H	0.9800
C14'—H14D	0.9800	C14A—H14I	0.9800
C14'—H14E	0.9800	C14"—H14J	0.9800
C14'—H14F	0.9800	C14"—H14K	0.9800
C17—C18	1.524 (9)	C14"—H14L	0.9800
C17—C19	1.536 (9)	C17A—C18A	1.514 (8)
C17—H17	1.0000	C17A—C19A	1.542 (9)
C18—H18A	0.9800	C17A—H17A	1.0000
C18—H18B	0.9800	C18A—H18D	0.9800
C18—H18C	0.9800	C18A—H18E	0.9800
C19—H19A	0.9800	C18A—H18F	0.9800
C19—H19B	0.9800	C19A—H19D	0.9800
C19—H19C	0.9800	C19A—H19E	0.9800
O1A—C10C	1.447 (6)	C19A—H19F	0.9800
			
C10—O1—O2	114.3 (4)	C7A—O2A—O1A	112.8 (4)
C7—O2—O1	111.6 (3)	C13A—O3A—C12A	116.5 (8)
C13—O3—C12	118.3 (5)	C13"—O3"—C12A	118.9 (12)
C15—O6—H6	109.5	C15A—O6A—H6A	109.5
C10B—C1—C2	105.8 (5)	C10E—C1A—C2A	105.6 (4)
C10B—C1—H1A	110.6	C10E—C1A—H1A1	110.6
C2—C1—H1A	110.6	C2A—C1A—H1A1	110.6
C10B—C1—H1B	110.6	C10E—C1A—H1A2	110.6
C2—C1—H1B	110.6	C2A—C1A—H1A2	110.6
H1A—C1—H1B	108.7	H1A1—C1A—H1A2	108.7
C1—C2—C3	107.7 (5)	C1A—C2A—C3B	106.1 (4)
C1—C2—H2A	110.2	C1A—C2A—H2A1	110.5
C3—C2—H2A	110.2	C3B—C2A—H2A1	110.5
C1—C2—H2B	110.2	C1A—C2A—H2A2	110.5
C3—C2—H2B	110.2	C3B—C2A—H2A2	110.5
H2A—C2—H2B	108.5	H2A1—C2A—H2A2	108.7
C17—C3—C2	113.9 (5)	C17A—C3B—C2A	113.5 (4)
C17—C3—C3A	119.8 (4)	C17A—C3B—C3C	119.2 (4)
C2—C3—C3A	101.9 (4)	C2A—C3B—C3C	103.8 (4)
C17—C3—H3	106.8	C17A—C3B—H3B	106.5
C2—C3—H3	106.8	C2A—C3B—H3B	106.5
C3A—C3—H3	106.8	C3C—C3B—H3B	106.5
C4—C3A—C15	111.1 (4)	C4A—C3C—C15A	110.6 (4)
C4—C3A—C10B	106.1 (5)	C4A—C3C—C10E	106.5 (4)
C15—C3A—C10B	112.6 (5)	C15A—C3C—C10E	113.4 (5)
C4—C3A—C3	117.5 (5)	C4A—C3C—C3B	118.1 (5)
C15—C3A—C3	108.5 (5)	C15A—C3C—C3B	107.9 (4)
C10B—C3A—C3	100.7 (4)	C10E—C3C—C3B	100.1 (4)
C3A—C4—C5	109.2 (5)	C3C—C4A—C5B	109.4 (5)
C3A—C4—H4A	109.8	C3C—C4A—H4A1	109.8
C5—C4—H4A	109.8	C5B—C4A—H4A1	109.8
C3A—C4—H4B	109.8	C3C—C4A—H4A2	109.8
C5—C4—H4B	109.8	C5B—C4A—H4A2	109.8
H4A—C4—H4B	108.3	H4A1—C4A—H4A2	108.2
C4—C5—C5A	118.2 (4)	C4A—C5B—C5C	118.2 (5)
C4—C5—H5A	107.8	C4A—C5B—H5B1	107.8
C5A—C5—H5A	107.8	C5C—C5B—H5B1	107.8
C4—C5—H5B	107.8	C4A—C5B—H5B2	107.8
C5A—C5—H5B	107.8	C5C—C5B—H5B2	107.8
H5A—C5—H5B	107.1	H5B1—C5B—H5B2	107.1
C6—C5A—C12	107.1 (5)	C12A—C5C—C5B	104.7 (4)
C6—C5A—C5	109.0 (4)	C12A—C5C—C6A	107.7 (4)
C12—C5A—C5	103.9 (4)	C5B—C5C—C6A	109.4 (4)
C6—C5A—C10A	111.7 (4)	C12A—C5C—C10D	113.0 (5)
C12—C5A—C10A	112.7 (4)	C5B—C5C—C10D	111.5 (4)
C5—C5A—C10A	112.0 (5)	C6A—C5C—C10D	110.4 (4)
C5A—C6—C7	119.2 (5)	C7A—C6A—C5C	119.3 (5)
C5A—C6—H6C	107.5	C7A—C6A—H6A1	107.5
C7—C6—H6C	107.5	C5C—C6A—H6A1	107.5
C5A—C6—H6D	107.5	C7A—C6A—H6A2	107.5
C7—C6—H6D	107.5	C5C—C6A—H6A2	107.5
H6C—C6—H6D	107.0	H6A1—C6A—H6A2	107.0
O2—C7—C8	107.8 (5)	O2A—C7A—C8A	109.6 (5)
O2—C7—C6	110.7 (5)	O2A—C7A—C6A	111.0 (5)
C8—C7—C6	117.5 (4)	C8A—C7A—C6A	115.5 (5)
O2—C7—H7	106.7	O2A—C7A—H7A	106.7
C8—C7—H7	106.7	C8A—C7A—H7A	106.7
C6—C7—H7	106.7	C6A—C7A—H7A	106.7
C9—C8—C11	126.0 (6)	C9A—C8A—C7A	114.0 (5)
C9—C8—C7	113.1 (6)	C9A—C8A—C11A	125.3 (6)
C11—C8—C7	120.8 (6)	C7A—C8A—C11A	120.7 (5)
C8—C9—C10	117.5 (6)	C8A—C9A—C10C	116.6 (6)
C8—C9—H9	121.2	C8A—C9A—H9A	121.7
C10—C9—H9	121.2	C10C—C9A—H9A	121.7
C10B—C10A—C10	107.6 (4)	O1A—C10C—C9A	108.9 (4)
C10B—C10A—C5A	112.6 (5)	O1A—C10C—C10D	111.7 (4)
C10—C10A—C5A	115.4 (5)	C9A—C10C—C10D	116.0 (5)
C10B—C10A—H10A	106.9	O1A—C10C—H10C	106.5
C10—C10A—H10A	106.9	C9A—C10C—H10C	106.5
C5A—C10A—H10A	106.9	C10D—C10C—H10C	106.5
C1—C10B—C10A	119.0 (5)	C10E—C10D—C10C	109.0 (4)
C1—C10B—C3A	105.5 (5)	C10E—C10D—C5C	112.3 (5)
C10A—C10B—C3A	114.9 (4)	C10C—C10D—C5C	116.0 (4)
C1—C10B—H10B	105.4	C10E—C10D—H10D	106.3
C10A—C10B—H10B	105.4	C10C—C10D—H10D	106.3
C3A—C10B—H10B	105.4	C5C—C10D—H10D	106.3
O1—C10—C9	109.7 (4)	C10D—C10E—C1A	117.9 (5)
O1—C10—C10A	111.7 (5)	C10D—C10E—C3C	115.0 (4)
C9—C10—C10A	115.1 (4)	C1A—C10E—C3C	106.3 (4)
O1—C10—H10	106.6	C10D—C10E—H10E	105.5
C9—C10—H10	106.6	C1A—C10E—H10E	105.5
C10A—C10—H10	106.6	C3C—C10E—H10E	105.5
C8—C11—H11A	109.5	C8A—C11A—H11D	109.5
C8—C11—H11B	109.5	C8A—C11A—H11E	109.5
H11A—C11—H11B	109.5	H11D—C11A—H11E	109.5
C8—C11—H11C	109.5	C8A—C11A—H11F	109.5
H11A—C11—H11C	109.5	H11D—C11A—H11F	109.5
H11B—C11—H11C	109.5	H11E—C11A—H11F	109.5
O3—C12—C5A	107.8 (5)	O3A—C12A—O3"	30.3 (4)
O3—C12—H12A	110.2	O3A—C12A—C5C	108.2 (4)
C5A—C12—H12A	110.2	O3"—C12A—C5C	112.1 (6)
O3—C12—H12B	110.2	O3A—C12A—H12C	110.1
C5A—C12—H12B	110.2	O3"—C12A—H12C	130.0
H12A—C12—H12B	108.5	C5C—C12A—H12C	110.1
O4'—C13—O3	120.2 (9)	O3A—C12A—H12D	110.1
O4'—C13—O4	38.7 (7)	O3"—C12A—H12D	81.0
O3—C13—O4	121.5 (7)	C5C—C12A—H12D	110.1
O4'—C13—C14	116.2 (9)	H12C—C12A—H12D	108.4
O3—C13—C14	113.8 (8)	O4A—C13A—O3A	122.1 (11)
O4—C13—C14	123.9 (9)	O4A—C13A—C14A	121.1 (13)
O4'—C13—C14'	126.0 (10)	O3A—C13A—C14A	116.8 (11)
O3—C13—C14'	112.2 (8)	O4"—C13"—O3"	119.9 (14)
O4—C13—C14'	118.1 (9)	O4"—C13"—C14"	125.0 (16)
C14—C13—C14'	20.3 (7)	O3"—C13"—C14"	114.0 (16)
C13—C14—H14A	109.5	C13A—C14A—H14G	109.5
C13—C14—H14B	109.5	C13A—C14A—H14H	109.5
H14A—C14—H14B	109.5	H14G—C14A—H14H	109.5
C13—C14—H14C	109.5	C13A—C14A—H14I	109.5
H14A—C14—H14C	109.5	H14G—C14A—H14I	109.5
H14B—C14—H14C	109.5	H14H—C14A—H14I	109.5
C13—C14'—H14D	109.5	C13"—C14"—H14J	109.5
C13—C14'—H14E	109.5	C13"—C14"—H14K	109.5
H14D—C14'—H14E	109.5	H14J—C14"—H14K	109.5
C13—C14'—H14F	109.5	C13"—C14"—H14L	109.5
H14D—C14'—H14F	109.5	H14J—C14"—H14L	109.5
H14E—C14'—H14F	109.5	H14K—C14"—H14L	109.5
O5—C15—O6	121.8 (5)	O5A—C15A—O6A	121.3 (5)
O5—C15—C3A	122.7 (5)	O5A—C15A—C3C	122.4 (5)
O6—C15—C3A	115.4 (5)	O6A—C15A—C3C	116.3 (5)
C3—C17—C18	114.2 (5)	C18A—C17A—C3B	113.5 (5)
C3—C17—C19	110.5 (4)	C18A—C17A—C19A	108.6 (5)
C18—C17—C19	108.5 (6)	C3B—C17A—C19A	110.6 (4)
C3—C17—H17	107.8	C18A—C17A—H17A	108.0
C18—C17—H17	107.8	C3B—C17A—H17A	108.0
C19—C17—H17	107.8	C19A—C17A—H17A	108.0
C17—C18—H18A	109.5	C17A—C18A—H18D	109.5
C17—C18—H18B	109.5	C17A—C18A—H18E	109.5
H18A—C18—H18B	109.5	H18D—C18A—H18E	109.5
C17—C18—H18C	109.5	C17A—C18A—H18F	109.5
H18A—C18—H18C	109.5	H18D—C18A—H18F	109.5
H18B—C18—H18C	109.5	H18E—C18A—H18F	109.5
C17—C19—H19A	109.5	C17A—C19A—H19D	109.5
C17—C19—H19B	109.5	C17A—C19A—H19E	109.5
H19A—C19—H19B	109.5	H19D—C19A—H19E	109.5
C17—C19—H19C	109.5	C17A—C19A—H19F	109.5
H19A—C19—H19C	109.5	H19D—C19A—H19F	109.5
H19B—C19—H19C	109.5	H19E—C19A—H19F	109.5
C10C—O1A—O2A	113.8 (4)		
			
C10—O1—O2—C7	10.9 (5)	C1A—C2A—C3B—C3C	26.5 (5)
C10B—C1—C2—C3	−3.8 (6)	C17A—C3B—C3C—C4A	77.4 (6)
C1—C2—C3—C17	158.9 (4)	C2A—C3B—C3C—C4A	−155.2 (4)
C1—C2—C3—C3A	28.4 (5)	C17A—C3B—C3C—C15A	−48.8 (6)
C17—C3—C3A—C4	77.6 (6)	C2A—C3B—C3C—C15A	78.6 (5)
C2—C3—C3A—C4	−155.7 (4)	C17A—C3B—C3C—C10E	−167.5 (5)
C17—C3—C3A—C15	−49.4 (7)	C2A—C3B—C3C—C10E	−40.2 (5)
C2—C3—C3A—C15	77.3 (5)	C15A—C3C—C4A—C5B	−63.1 (5)
C17—C3—C3A—C10B	−167.7 (5)	C10E—C3C—C4A—C5B	60.5 (5)
C2—C3—C3A—C10B	−41.0 (5)	C3B—C3C—C4A—C5B	172.0 (4)
C15—C3A—C4—C5	−62.5 (6)	C3C—C4A—C5B—C5C	−53.5 (6)
C10B—C3A—C4—C5	60.2 (5)	C4A—C5B—C5C—C12A	162.7 (5)
C3—C3A—C4—C5	171.8 (4)	C4A—C5B—C5C—C6A	−82.1 (6)
C3A—C4—C5—C5A	−53.1 (6)	C4A—C5B—C5C—C10D	40.2 (6)
C4—C5—C5A—C6	−84.2 (6)	C12A—C5C—C6A—C7A	−78.7 (6)
C4—C5—C5A—C12	161.9 (5)	C5B—C5C—C6A—C7A	168.1 (5)
C4—C5—C5A—C10A	40.0 (6)	C10D—C5C—C6A—C7A	45.1 (7)
C12—C5A—C6—C7	−76.6 (6)	O1A—O2A—C7A—C8A	−52.1 (5)
C5—C5A—C6—C7	171.6 (5)	O1A—O2A—C7A—C6A	76.7 (5)
C10A—C5A—C6—C7	47.3 (7)	C5C—C6A—C7A—O2A	−89.1 (6)
O1—O2—C7—C8	−58.8 (5)	C5C—C6A—C7A—C8A	36.5 (7)
O1—O2—C7—C6	71.0 (5)	O2A—C7A—C8A—C9A	51.0 (6)
C5A—C6—C7—O2	−92.1 (6)	C6A—C7A—C8A—C9A	−75.4 (7)
C5A—C6—C7—C8	32.3 (8)	O2A—C7A—C8A—C11A	−130.5 (5)
O2—C7—C8—C9	53.4 (6)	C6A—C7A—C8A—C11A	103.2 (6)
C6—C7—C8—C9	−72.5 (6)	C7A—C8A—C9A—C10C	1.3 (7)
O2—C7—C8—C11	−128.9 (5)	C11A—C8A—C9A—C10C	−177.1 (6)
C6—C7—C8—C11	105.2 (6)	O2A—O1A—C10C—C9A	47.6 (6)
C11—C8—C9—C10	−176.6 (5)	O2A—O1A—C10C—C10D	−81.8 (5)
C7—C8—C9—C10	0.9 (7)	C8A—C9A—C10C—O1A	−51.0 (7)
C6—C5A—C10A—C10B	85.2 (6)	C8A—C9A—C10C—C10D	76.0 (6)
C12—C5A—C10A—C10B	−154.2 (4)	O1A—C10C—C10D—C10E	−41.8 (6)
C5—C5A—C10A—C10B	−37.5 (6)	C9A—C10C—C10D—C10E	−167.4 (5)
C6—C5A—C10A—C10	−39.0 (7)	O1A—C10C—C10D—C5C	86.2 (6)
C12—C5A—C10A—C10	81.7 (5)	C9A—C10C—C10D—C5C	−39.4 (7)
C5—C5A—C10A—C10	−161.6 (4)	C12A—C5C—C10D—C10E	−155.1 (4)
C2—C1—C10B—C10A	−153.8 (4)	C5B—C5C—C10D—C10E	−37.5 (6)
C2—C1—C10B—C3A	−22.9 (5)	C6A—C5C—C10D—C10E	84.2 (5)
C10—C10A—C10B—C1	−53.1 (6)	C12A—C5C—C10D—C10C	78.6 (5)
C5A—C10A—C10B—C1	178.6 (4)	C5B—C5C—C10D—C10C	−163.8 (4)
C10—C10A—C10B—C3A	−179.5 (4)	C6A—C5C—C10D—C10C	−42.1 (6)
C5A—C10A—C10B—C3A	52.2 (6)	C10C—C10D—C10E—C1A	−51.2 (6)
C4—C3A—C10B—C1	163.0 (4)	C5C—C10D—C10E—C1A	178.8 (4)
C15—C3A—C10B—C1	−75.2 (6)	C10C—C10D—C10E—C3C	−178.0 (4)
C3—C3A—C10B—C1	40.0 (5)	C5C—C10D—C10E—C3C	52.0 (6)
C4—C3A—C10B—C10A	−63.8 (6)	C2A—C1A—C10E—C10D	−155.3 (4)
C15—C3A—C10B—C10A	57.9 (6)	C2A—C1A—C10E—C3C	−24.5 (5)
C3—C3A—C10B—C10A	173.2 (5)	C4A—C3C—C10E—C10D	−63.8 (6)
O2—O1—C10—C9	41.4 (6)	C15A—C3C—C10E—C10D	58.1 (6)
O2—O1—C10—C10A	−87.5 (5)	C3B—C3C—C10E—C10D	172.7 (4)
C8—C9—C10—O1	−49.3 (6)	C4A—C3C—C10E—C1A	163.7 (4)
C8—C9—C10—C10A	77.7 (6)	C15A—C3C—C10E—C1A	−74.4 (5)
C10B—C10A—C10—O1	−44.8 (6)	C3B—C3C—C10E—C1A	40.2 (5)
C5A—C10A—C10—O1	81.8 (6)	C13A—O3A—C12A—O3"	−0.6 (11)
C10B—C10A—C10—C9	−170.8 (5)	C13A—O3A—C12A—C5C	−103.2 (9)
C5A—C10A—C10—C9	−44.2 (7)	C13"—O3"—C12A—O3A	34.6 (10)
C13—O3—C12—C5A	−144.2 (6)	C13"—O3"—C12A—C5C	123.2 (11)
C6—C5A—C12—O3	175.9 (4)	C5B—C5C—C12A—O3A	−56.5 (6)
C5—C5A—C12—O3	−68.8 (5)	C6A—C5C—C12A—O3A	−172.8 (5)
C10A—C5A—C12—O3	52.7 (6)	C10D—C5C—C12A—O3A	65.0 (6)
C12—O3—C13—O4'	−29.3 (14)	C5B—C5C—C12A—O3"	−88.6 (7)
C12—O3—C13—O4	16.1 (12)	C6A—C5C—C12A—O3"	155.1 (6)
C12—O3—C13—C14	−173.9 (10)	C10D—C5C—C12A—O3"	32.9 (7)
C12—O3—C13—C14'	164.1 (10)	C12A—O3A—C13A—O4A	0.2 (16)
C4—C3A—C15—O5	139.8 (6)	C12A—O3A—C13A—C14A	−177.1 (9)
C10B—C3A—C15—O5	20.9 (8)	C12A—O3"—C13"—O4"	7(2)
C3—C3A—C15—O5	−89.6 (7)	C12A—O3"—C13"—C14"	−161.5 (14)
C4—C3A—C15—O6	−44.8 (7)	C4A—C3C—C15A—O5A	141.8 (5)
C10B—C3A—C15—O6	−163.7 (5)	C10E—C3C—C15A—O5A	22.2 (7)
C3—C3A—C15—O6	85.9 (6)	C3B—C3C—C15A—O5A	−87.7 (6)
C2—C3—C17—C18	177.3 (5)	C4A—C3C—C15A—O6A	−41.2 (6)
C3A—C3—C17—C18	−61.7 (7)	C10E—C3C—C15A—O6A	−160.8 (5)
C2—C3—C17—C19	54.7 (7)	C3B—C3C—C15A—O6A	89.3 (5)
C3A—C3—C17—C19	175.7 (6)	C2A—C3B—C17A—C18A	174.7 (5)
C10C—O1A—O2A—C7A	2.6 (5)	C3C—C3B—C17A—C18A	−62.5 (7)
C10E—C1A—C2A—C3B	−1.5 (5)	C2A—C3B—C17A—C19A	52.4 (6)
C1A—C2A—C3B—C17A	157.3 (4)	C3C—C3B—C17A—C19A	175.1 (5)

### Hydrogen-bond geometry (Å, °)

**Table:**

*D*—H···*A*	*D*—H	H···*A*	*D*···*A*	*D*—H···*A*
O6—H6···O5A	0.84	1.90	2.714 (5)	162.
O6A—H6A···O5	0.84	1.88	2.690 (5)	161.
C1A—H1A2···O5A	0.99	2.59	2.972 (9)	103
C5—H5B···O3	0.99	2.39	2.893 (10)	110
C5B—H5B1···O4A^i^	0.99	2.37	3.257 (14)	149
C5B—H5B2···O3A	0.99	2.22	2.751 (13)	112
C10—H10···O2^ii^	1.00	2.34	3.259 (9)	152
C10A—H10A···O3	1.00	2.39	2.894 (6)	111
C10A—H10A···O5	1.00	2.50	3.134 (9)	121
C10B—H10B···O1	1.00	2.41	2.763 (9)	100
C10C—H10C···O2A^iii^	1.00	2.46	3.350 (8)	147
C10D—H10D···O3A	1.00	2.56	3.016 (12)	107
C10D—H10D···O5A	1.00	2.51	3.151 (8)	122
C12—H12B···O4	0.99	2.28	2.691 (17)	103
C12A—H12D···O4A	0.99	2.21	2.623 (14)	103

Symmetry codes: (i) *x*, *y*−1, *z*; (ii) −*x*, *y*−1/2, −*z*+1; (iii) −*x*+1, *y*+1/2, −*z*.
